# Efficacy of wound care ointment in healing infectious wounds after anorectal surgery: A randomized controlled trial

**DOI:** 10.1097/MD.0000000000041347

**Published:** 2025-01-24

**Authors:** Lelai Yu, Yinfu Lei, Ke Yuan

**Affiliations:** a Anorectal Department, People’s Hospital of Leshan, Leshan, Sichuan, China.

**Keywords:** anorectal surgery, granulation, healing, infectious wound, wound care ointment

## Abstract

**Background:**

This study evaluates the efficacy of a novel bismuth subgallate-borneol compound ointment as an adjuvant therapy in promoting postoperative healing of infectious incisions after anorectal surgery.

**Methods:**

From June 2023 to October 2023, 46 patients with perianal abscess and anal fistula treated at our institution’s Anorectal Surgery Department were enrolled in this prospective randomized controlled study. Patients were randomly allocated into 2 groups: the experimental group (n = 23) received conventional wound care plus a proprietary ointment containing 4.5% bismuth subgallate and 0.7% d-borneol in a Vaseline base, while the control group (n = 23 cases) received conventional wound care alone (comprising daily wound cleansing, dressing changes and traditional Chinese medicine injection). Image J Software was used for collecting the data of wound area, and the wound healing rate and granulation growth rate of the 2 groups were calculated to evaluate the therapeutic effect of the 2 groups.

**Results:**

The growth rate of cured granulation in the experimental group was higher than that in the control group at all 3 predetermined observation points, with statistically significant difference (*P* < .05). Furthermore, the wound healing rate of the experimental group showed significant improvement by day 14 (*P* < .05).

**Conclusion:**

The bismuth subgallate-borneol compound ointment, when used as an adjuvant to standard wound care protocols, demonstrates significant efficacy in treating post-anorectal surgery infectious wounds. Its dual-active component formulation appears to effectively promote both granulation tissue formation and wound healing, potentially through its documented antibacterial properties.

## 1. Introduction

Anorectal diseases include infectious diseases, such as perianal abscess and anal fistula. This type of disease manifests as the formation of pus within the interstitial spaces among the perianal tissues due to bacterial invasion, resulting in the formation of an abscess cavity. Perianal abscess and anal fistula are different stages of this type of infectious disease. Anal fistula refers to the epithelialized canal formed between anal canal and skin around anus. At present, it is recognized that the etiology is caused by chronic infection and epithelialization of drainage canal of perianal abscess. The treatment for perianal abscess involves incision and drainage. Once diagnosed with perianal abscess, prompt incision and drainage should be performed.^[1,2]^ The surgical method involves incising the abscess cavity and fistula to ensure adequate drainage of pus. Necrotic tissue within the abscess cavity sloughs off, and granulation tissue fills the incision. Subsequently, surface skin tissue covers the wound and grows, allowing for wound healing. Over time, due to the defecation function of the anus, the wound is prone to fecal contamination, and since the incision is not routinely sutured, prolonged dressing changes are required for treatment. There are some difficulties in clinical work, such as long healing time, obvious pain in dressing change and troublesome dressing change operation. Rapid healing of wounds through drugs is the key to solve the above problems. In previous studies, there are literatures suggesting that Suile has a good effect on wound healing in patients with diabetes and burn.^[3]^ The surgical wounds of anorectal infectious diseases are similar to those of burn and diabetes related diseases, and there are necrotic tissues on the surface, so this drug is applied to anorectal infectious diseases. The wound care ointment contains 4.5% bismuth subgallate and 0.7% d-borneol, with Vaseline as the base, possessing antibacterial properties. It also exhibits anti-infective properties while maintaining a moist environment at the wound site. Furthermore, it can alleviate oxidative stress reactions, reduce tissue infiltration by inflammatory substances, accelerate granulation tissue formation, and promote wound healing. This study compares the therapeutic effects of using and not using the wound care ointment on infectious wounds after anorectal surgery. The aim is to provide clinical guidance for the healing of infectious wounds after anorectal surgery and the selection of appropriate medications. The following report is provided.

## 2. Data and methods

### 2.1. General information

From June 2023 to October 2023, 44 cases of perianal abscess and anal fistula in the Anorectal Surgery of our hospital were selected as the research objects. The sample size was calculated by G*power, repeated measures, *F*-tests, ANOVA: Repeated measures, with-between interaction, A priori: Compute required sample size-given α, power, and effect size; power: 0.95; Number of groups: 2; Number of measurements: 3, resulting in an overall sample size of 44. The last patient in the experimental group (n = 23 cases, plus wound care ointment on the basis of routine treatment) completed the last data collection on August 15th, and the last patient in the control group (n = 23 cases, routine treatment) completed the last data collection on October 7th. There was no significant difference in general data between the 2 groups (*P* > .05), but it was comparable. This study has been approved by the ethics committee of the author’s hospital.

### 2.2. Inclusion criteria

Adherence to the diagnostic criteria for perianal abscess and anal fistula as outlined in the “Chinese Expert Consensus on the Clinical Diagnosis and Treatment of Perianal Abscess” and “Chinese Expert Consensus on the Diagnosis and Treatment of Anal Fistula (2020 Edition)”^[4,5]^; surgical approach involving radical cure by incision and drainage with internal opening thread-drawing; patients providing informed consent and signing informed consent forms, and cooperating with the treatment;

### 2.3. Exclusion criteria

Individuals with malnutrition; those with concurrent tumors, autoimmune disorders, or bleeding disorders; patients with diabetes mellitus.

### 2.4. Methods

#### 2.4.1. Wound care

Both groups were treated with antibiotics for 3 days. Daily dressing changes required patients to clean the wound 3 times a day, wash the incision with clear water to clean the anus every time, and then spray povidone-iodine solution with a watering can. Daily, physicians changed the dressing once, utilizing povidone-iodine swabsticks to thoroughly clean the incision. Wound data was collected based on observation time points.

#### 2.4.2. Control group

Daily, physicians changed the dressing once, utilizing povidone-iodine swabsticks to thoroughly clean the incision, and then injected hemorrhoid antibacterial gel (Jilin Qiwei Biotechnology Co., Ltd., Jilin Medical Device Registration No. 20212140521) into the annus. Patients were required to clean the wound 3 times a day, wash the incision with clear water to clean the anus every time, spray povidone-iodine solution with a watering can, and then injected Fuzhiqing ointment into the annus. Then the surface was covered with clean gauze. The management protocol for the control group was established in accordance with 2 authoritative Chinese guidelines: “Expert Consensus on Clinical Pathway of Colorectal Surgery (2022)” and “Expert Consensus on Management of Perianal Infectious Wounds (2023 Edition).”

#### 2.4.3. Test group

Daily, physicians changed the dressing once, utilizing povidone-iodine swabsticks to thoroughly clean the incision, and then injected hemorrhoid antibacterial gel into the annus. Patients were required to clean the wound 3 times a day, wash the incision with clear water to clean the anus every time, and then spray povidone-iodine solution with a watering can. The patient was treated with wound care ointment (Beijing Baili Tiancheng Science and Trade Co., Ltd., National Medical Device Registration No. 20183640068). Approximately 2 cm length of the dressing was squeezed out and directly applied to the incision wound, followed by covering the surface with clean gauze. This procedure continued until the completion of the data collection period.

#### 2.4.4. Treatment precautions

Both groups were treated continuously until the wound healing. During the treatment, instructions were provided to the patients regarding precautions, ensuring patient compliance. During the wound healing period, both groups of patients received nutritional support and were encouraged to maintain a normal diet. Patients were also encouraged to walk to facilitate the drainage of pus and the shedding of necrotic tissue.

### 2.5. Observation indicators

#### 2.5.1. Wound recovery

Measurements were taken 4 times during the treatment period, on the second day post-surgery, the fourth day post-surgery, the seventh day post-surgery, and the fourteenth day post-surgery. Methods: The scale containing patient information was placed near the wound surface, and pictures were taken by mobile phone. After the pictures were collected, the wound area was calculated by using Image J software. During the observation process, artificial marking was performed on the growing granulation tissue, and the area was calculated. The growth rate of granulation tissue was then compared relative to the wound area. Then the change in wound area was measured and compared to the initial data to calculate wound healing rate. Subjective evaluation indicators from patients were not collected in this study.

### 2.6. Statistical analysis

SPSS 27.0 (Chicago) was used to analyze the data; Measurement data were presented as mean (±standard deviation) and analyzed using paired-sample *t*-test for independent samples. Data involving repeated measurements were subjected to repeated measures analysis of variance (ANOVA). *P* < .05 indicates a statistically significant difference.

## 3. Results

### 3.1. Comparison of wound recovery between 2 groups of patients

The granulation growth rate in the experimental group was consistently higher than that in the control group at 3 observation time points (*P* < .05), as shown in Table 1; there was no significant difference in the wound healing rate between the experimental and control groups. On the 4th and 7th days (*P* > .05). However, on the 14th day, the wound healing rate in the experimental group was significantly higher than that in the control group (*P* < .05), as shown in Table 2.

**Table 1:** Comparison of granulation growth rate between the 2 groups.

Granulation growth rate	Mean	Standard deviation	Mean difference	*t*	*P*
The 4th day of the experimental group	32.27	5.59	11.91	6.31	.000
The 4th day of the control group	20.36	7.25			
The 7th day of the experimental group	46.4	5.53	14.54	8.325	.000
The 7th day of the control group	31.86	7.48			
The 14th day of the experimental group	66.27	7.51	16.7	6.556	.000
The 14th day of the control group	49.57	9.57			

**Table 2:** Comparison of incision healing rate between the 2 groups.

Incision healing rate	Mean	Standard deviation	Mean difference	*t*	*P*
The 4th day of the experimental group	12.37	9.79	−0.9	−0.341	.736
The 4th day of the control group	13.27	5.56			
The 7th day of the experimental group	29.13	13.12	3.42	1.088	.289
The 7th day of the control group	25.71	5.84			
The 14th day of the experimental group	49.53	15.08	8.22	2.146	.044
The 14th day of the control group	41.31	5.89			

## 4. Discussion

**Figure 1.**
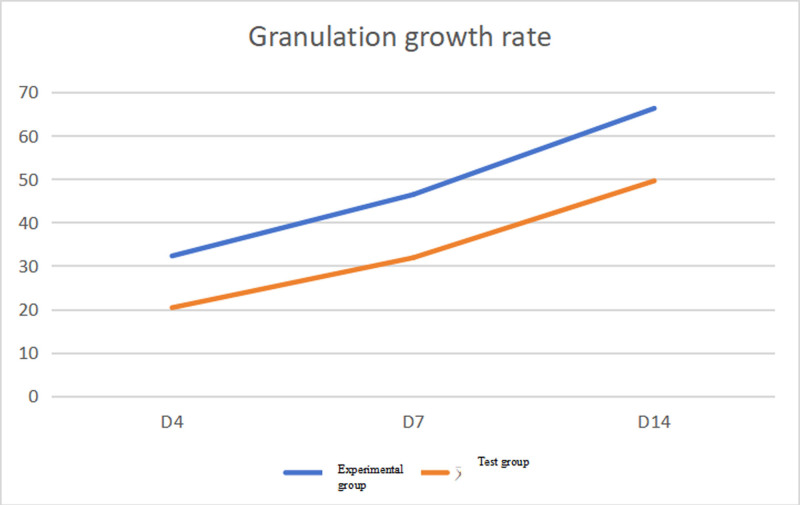
Trend of granulation growth rate.

In this study, after applying the bismuth subgallate-borneol compound ointment, the granulation growth rate in the experimental group was consistently higher than that in the control group at all 3 observation points (Fig. 1), with significant differences observed. This finding aligns with several previous studies. Research dating back to 2003^[6]^ established the beneficial interaction between bismuth subgallate and borneol, demonstrating their combined efficacy in accelerating wound healing processes of skin. Subsequently, Serena et al^[7]^ demonstrated that bismuth subgallate-borneol preparations exhibited superior efficacy in promoting acute wound healing compared to bacitracin.

**Figure 2.**
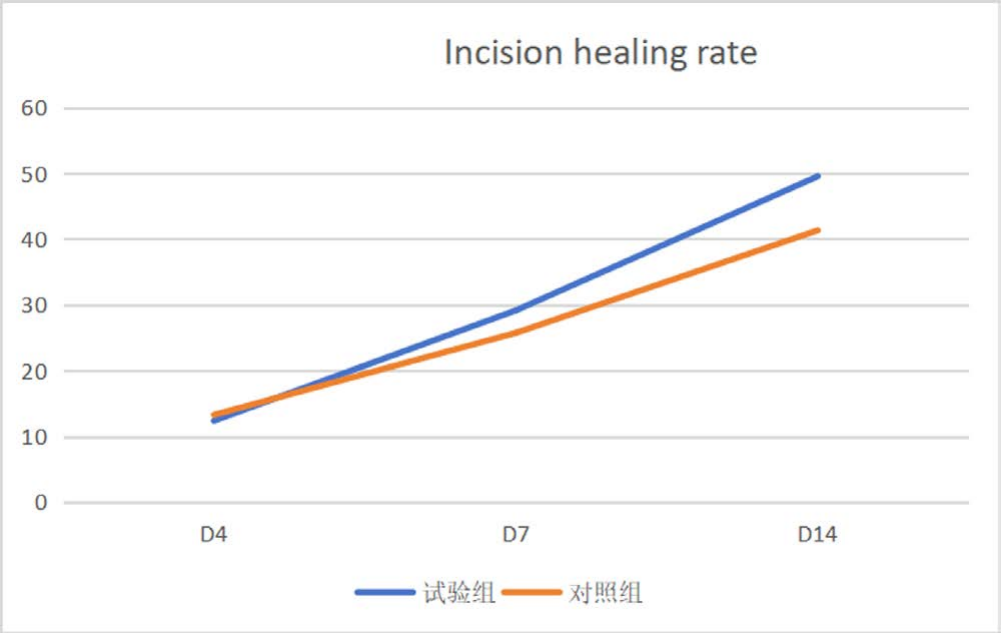
Trend of incision healing rate.

These indicate that the wound care ointment can effectively promote granulation tissue growth at the incision site. This may be attributed to maintaining a moist environment for the incision, inhibiting bacterial proliferation, reducing wound infection, promoting fibroblast growth, and facilitating granulation tissue formation. During days 4 to 7 of wound healing, there was a trend of superior healing rate, and by day 14, the data showed statistically significant differences between the 2 groups (Fig. 2). This is mainly because during the first week of wound healing, there is a period of pus discharge and necrotic tissue shedding, and the advantages of using wound care ointment become more pronounced as the wound enters the growth phase in the second week. These mechanisms have been well-documented in previous research.^[8]^

During days 4 to 7 of wound healing, we observed a trend of superior healing rate, and by day 14, the data showed statistically significant differences between the 2 groups (Fig. 2). The delayed manifestation of significant differences is primarily because during the first week of wound healing, there is a period of pus discharge and necrotic tissue shedding. This observation is supported by Almadani et al,^[9]^ who documented similar wound healing phases in their systematic review of wound healing.

**Figure 3.**
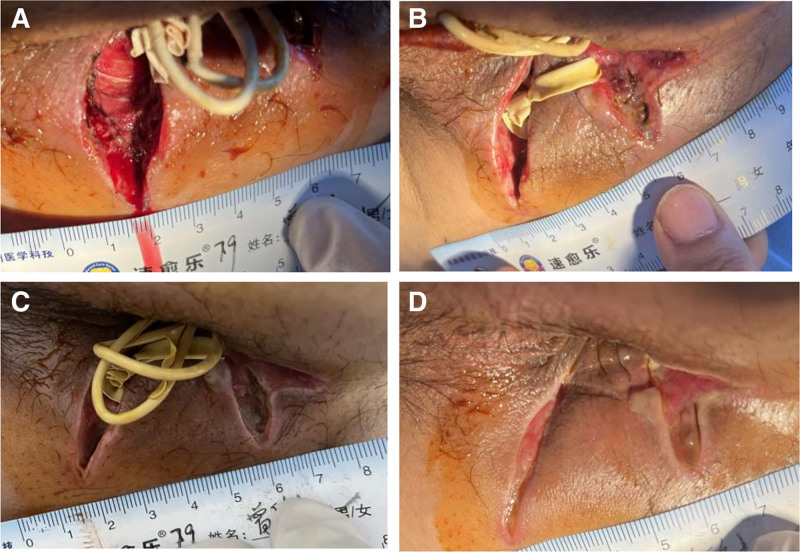
A 54-yr-old male was diagnosed with complex anal fistula combined abscess. (A) Taken the day after surgery following removal of the gauze showed a wound area of 4.85 cm², (B) captured on the fourth day after surgery, measured the wound area to be 3.957 cm², (C) captured at this time, measured the wound area to be 2.781 cm². (D) Taken at this stage, measured the wound area to be 1.553 cm².

Typical case: A 54-year-old male was diagnosed with complex anal fistula combined abscess. Before treatment, Figure 3A taken the day after surgery following removal of the gauze showed a wound area of 4.85 cm², with some of the wound tissue appearing fresh, while necrotic tissue was visible on the posterior aspect of the wound. Figure 3B, captured on the fourth day after surgery, measured the wound area to be 3.957 cm², with granulation tissue beginning to form and necrotic tissue gradually sloughing off. Seven days after surgery, granulation tissue continued to proliferate, and the wound contracted, showing signs of skin growth along the edges. Figure 3C, captured at this time, measured the wound area to be 2.781 cm². Fourteen days post-surgery, the surgical incision was nearly filled with granulation tissue, and there was significant proliferation of skin tissue. Figure 3D, taken at this stage, measured the wound area to be 1.553 cm². The wound was essentially healed, without edema, and fresh red granulation tissue was growing well, with no exudate.

## 5. Conclusion

To sum up, the wound care ointment has shown significant effectiveness in promoting wound healing following perianal abscess and anal fistula surgeries. It enhances the growth rate of granulation tissue, facilitating wound recovery, and is worthy of clinical promotion.

## Acknowledgments

We would like to acknowledge the reviewers for their helpful comments on this paper.

## Author contributions

**Conceptualization:** Lelai Yu.

**Data curation:** Ke Yuan.

**Methodology:** Lelai Yu.

**Software:** Lelai Yu.

**Supervision:** Yinfu Lei.

**Writing – original draft:** Lelai Yu.
